# Deciphering vesicle-assisted transport mechanisms in cytoplasm to cilium trafficking

**DOI:** 10.3389/fncel.2024.1379976

**Published:** 2024-05-27

**Authors:** Mark Tingey, Andrew Ruba, Zechang Jiang, Weidong Yang

**Affiliations:** Department of Biology, Temple University, Philadelphia, PA, United States

**Keywords:** primary cilium, three-dimensional imaging (3D imaging), transformation algorithm, super-resolution, vesicle traffic, transition zone, intraflagellar transport, BBsome

## Abstract

The cilium, a pivotal organelle crucial for cell signaling and proper cell function, relies on meticulous macromolecular transport from the cytoplasm for its formation and maintenance. While the intraflagellar transport (IFT) pathway has traditionally been the focus of extensive study concerning ciliogenesis and ciliary maintenance, recent research highlights a complementary and alternative mechanism—vesicle-assisted transport (VAT) in cytoplasm to cilium trafficking. Despite its potential significance, the VAT pathway remains largely uncharacterized. This review explores recent studies providing evidence for the dynamics of vesicle-related diffusion and transport within the live primary cilium, employing high-speed super-resolution light microscopy. Additionally, we analyze the spatial distribution of vesicles in the cilium, mainly relying on electron microscopy data. By scrutinizing the VAT pathways that facilitate cargo transport into the cilium, with a specific emphasis on recent advancements and imaging data, our objective is to synthesize a comprehensive model of ciliary transport through the integration of IFT-VAT mechanisms.

## 1 Introduction

Projecting from the plasma membrane of nearly all vertebrate cell types are antennae like projections termed primary cilium. These evolutionarily conserved organelles function generally as sensors through which cells receive signals from light, chemical, or mechanical stimuli enabling cell function and signal transduction (Nauli et al., 2003; Corbit et al., 2005; Satir and Christensen, 2007; Hsiao et al., 2012). In fact, the primary cilium has been identified as an important component of key signaling pathways, including the Sonic hedgehog and Wnt Pathways (Hassounah et al., 2017; Lee, 2020; Liu et al., 2020; Zhang et al., 2023). In light of these roles, it is unsurprising that disruption of the primary cilium is associated with a wide variety of disorders, referred to commonly as ciliopathies. These disorders include renal disease, retinal degeneration, hearing loss, anosmia a variety of cancers, laterality defects, intellectual disability, obesity, polydactyly, and skeletal abnormalities (Mitchison and Valente, 2017; Reiter and Leroux, 2017; Andreu-Cervera et al., 2021). Of particular interest is the role played by primary cilia in neurological diseases such as Joubert syndrome, Bardet-Biedl Syndrome, Meckel-Gruber syndrome alongside an emerging role in neurodegenerative diseases such as Alzheimer’s Disease and Parkinson’s Disease (Karunakaran et al., 2020; Shamseldin et al., 2020; Ki et al., 2021; Mohieldin et al., 2021a; Ma et al., 2022). The consequential nature of this organelle has resulted in significant scientific interest in understanding both the structure and dynamics of the primary cilium, particularly as it relates to signaling.

**FIGURE 1:**
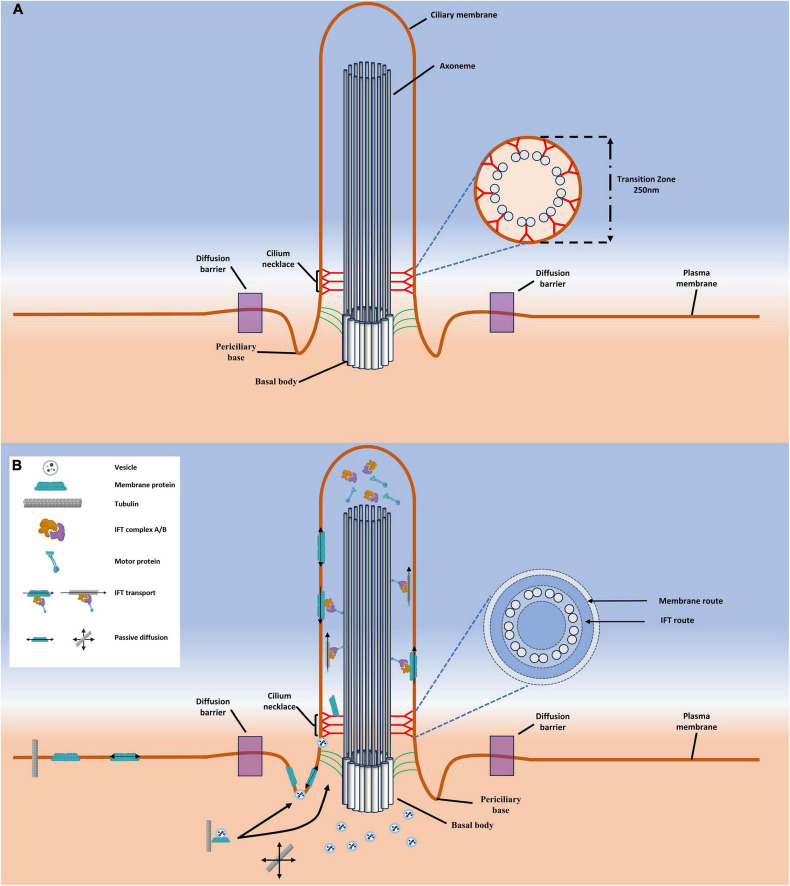
A schematic of generalized cilia structure highlighting import pathways of macromolecules. **(A)** Shown here are the structural components of the primary cilia, including the ciliary necklace, periciliary base, basal body, transition zone, and axoneme. **(B)** The currently accepted pathways for macromolecular transport into the cilium. Depicted here are two primary routes, the vesicle route that depicts a cargo bearing vesicle fusing at an unknown point between the diffusion barrier and the transition zone, and the IFT train. The IFT train assembles near the base of the cilium and then passes into the cilium along the IFT route via motor proteins.

The ultrastructure of the primary cilium consists of a finger-like structure that contains a ciliary skeleton composed of microtubules surrounded by a cell membrane (Figure 1A; Breslow et al., 2013; Ke and Yang, 2014). The primary cilium measures between 1 and 5 μm in length with a width of 0.2 μm, although the width decreases closer to the distal tip (Scherft and Daems, 1967). The primary cilium can be further divided into three discrete structural subsections: The basal body, the transition zone, and the axoneme. The basal body is composed of 9 triplets of gamma tubulin and serves as both the origination point and base of the primary cilium (Hoey et al., 2012; Kim and Dynlacht, 2013; Wheway et al., 2018). From the surface of the cell projects the body of the primary cilium, the axoneme. The axoneme of the primary cilium is supported by a microtubule-based skeleton consisting of nine peripheral doublets commonly termed as a 9 + 0 pattern (Kim and Dynlacht, 2013; Wang et al., 2020). This is in contrast to the microtubules found in motile cilia which contain a 9 + 2 pattern with nine peripheral and two central doublets (Eley et al., 2005; Hoey et al., 2012; Wang et al., 2020). Between the basal body and the axoneme is the transition zone of the primary cilium and is named such as at this region the triplet microtubules of the basal body transition to the doublet microtubules of the axoneme (Blasius et al., 2019). Intriguingly, while the primary cilium is recognized as a cellular organelle, it is not membrane bound. Indeed, a casual observation of the primary cilium could lead a researcher to conclude that movement between the cytoplasm and cilium is unregulated. Despite the lack of a membrane in this region, the transition zone serves as a selectively permeable barrier between the body of the primary cilium and the cytoplasm of the cell that is sometimes referred to as the ciliary gate (Szymanska and Johnson, 2012; Wu et al., 2012; Park and Leroux, 2022a).

The transition zone is best characterized by the presence of electron dense Y-shaped structures (Y-links) that span the region between the doublet microtubules and the ciliary membrane (Figure 1A; Takao and Verhey, 2016). Of some notable interest is the considerable degree of evolutionary conservation of the Y-links in primary cilium (Park and Leroux, 2022a). This structure has been observed within the transition zone of motile cilia of both *Chlamydomonas* (Craige et al., 2010) and rabbit oviducts (Anderson, 1974); as well as non-motile cilia from *C. elegans* (Jensen et al., 2018) and rat photoreceptors (Besharse et al., 1985). Such a high degree of conservation suggests that this structure plays a critical role in cell processes, an observation that bears out as dysregulation of transition zone proteins are commonly associated with ciliopathies (Barker et al., 2014; Dean et al., 2016; Epting et al., 2022).

A number of proteins have been identified as localizing to the transition zone, the majority of which were identified due to their role in human ciliopathies. These proteins have been found to exist in three functional modules: (1) the NPHP1/4 complex that contains NPHP1, NPHP4, and NPHP8, (2) the NPHP5/6 complex containing NPHP5 and NPHP6, and (3) the complex associating with the membrane termed the MKS complex containing MKS1, B9D1, B9D2, Tectonic1, Tectonic2, TMEM231, TMEM216, TMEM67, and MKS6 (Garcia-Gonzalo and Reiter, 2017; Gonçalves and Pelletier, 2017; Jensen and Leroux, 2017; Blasius et al., 2019). The membrane bound MKS complex associates via weak interactions mediated by CEP290 with the NPHP complexes that are bound to the microtubule doublet primarily by NPHP4 (Garcia-Gonzalo and Reiter, 2017), thereby creating superstructure that spans from the microtubule doublet of the axoneme to the membrane within the transition zone. NPHP4 is of particular interest within the transition zone proteins as NPHP4 localizes specifically to the transition zone (Omran, 2010; Shiba and Yokoyama, 2012; Garcia-Gonzalo and Reiter, 2017), has been demonstrated to regulate NPHP1 localization to the transition zone in both human retinal pigment epithelial cells and *C. elegans* sensory cilia (Winkelbauer et al., 2005; Liebau et al., 2011), facilitate direct interaction between NPHP1 and NPHP8 to form the NPHP1-4-8 complex (Sang et al., 2011; Shiba and Yokoyama, 2012), and have been shown to be involved in the regulation of both membrane and large soluble proteins at the transition zone (Awata et al., 2014). Together, this makes NPHP4 an ideal target for labeling in order to visualize the transition zone.

Cilia are found on both quiescent and proliferating cells in the G1 phase of the cell cycle (Shiba and Yokoyama, 2012) and are generated through a process termed ciliogenesis. Ciliogenesis occurs in several stages. First, the cell exits mitosis, freeing up the centriole to begin axoneme nucleation. Next, ciliary appendages are added, followed by the docking of the centriole to a post-Golgi vesicle, which then fuses with the plasma membrane. At this point, the centriole becomes known as the basal body. Lastly, the import of intraflagellar transport (IFT) associated proteins facilitates axoneme extension, resulting in a fully formed cilium (Winkelbauer et al., 2005; Liebau et al., 2011; Shiba and Yokoyama, 2012). This review will commence with an exploration of the role of the transition zone as the barrier to diffusion and the transport of cargoes across this selectively permeable membrane via IFT and other mechanisms. The focus will be on a recent study highlighting an expanded role for vesicles in cargo import.

## 2 Transition zone likely functions as a selectively permeable barrier

The idea of a structure regulating transport of macromolecules into the primary cilium was first proposed by Sjöstrand (1953) after observing via transmission electron microscopy (TEM) that there appeared to be a structure separating the cytoplasm from the body of the cilium. This observation was later supported through the recognition that the ciliary membrane has a distinct lipid composition, despite being contiguous with the cell’s plasma membrane. Further, analysis of soluble proteins within the cilium found a unique population when compared to that of the cell body indicating some form of selective ciliary import (Emmer et al., 2010; Nachury et al., 2010; Madhivanan and Aguilar, 2014; Takao and Verhey, 2016). The observation that both the body and membrane of the primary cilium have a distinct protein composition from that of the cytoplasm or plasma membrane, despite being continuous, leads to the question: does the primary cilium have two discrete barriers to transport, one between the body of the cilium and the cytoplasm, and another between the ciliary membrane and the cell’s plasma membrane? It appears that there are indeed two discrete barriers to transport, although how the transition zone proteins assemble in order to generate these two discrete barriers remains uncertain (Park and Leroux, 2022b).

While the exact mechanisms are unknown, it has been suggested that a series of proteins localizing to the base of the ciliary membrane called the ciliary necklace, directly distal to the ciliary pocket, may be the cause. As early as 1972, the ciliary necklace was proposed to serve as a barrier to membrane diffusion (Gilula and Satir, 1972; Montesano, 1979; Wingfield et al., 2018). This structure is thought to be the extracellular domains of Y-links in the transition zone that connect the axonemal doublets to the ciliary membrane (Blacque and Sanders, 2014). The membrane diffusion barrier is likely not only the result of the ciliary necklace, but a result of the ciliary pocket, necklace, and transitional fibers working in tandem. Indeed, it has been proposed that the highly curved nature of the membrane at the base of the pocket itself may impose a geometric constraint inhibiting free diffusion while the transitional fibers interacting with the membrane at the base of the ciliary membrane may also hinder diffusion (Rohatgi and Snell, 2010).

Regarding transport into the primary cilium from the cytoplasm of the cell, it has been suggested that the transition zone functions as a selectively permeable barrier similar to the Nuclear Pore Complex (NPC). The NPC, a selectively permeable barrier regulating import into- and export out of the nucleus, permits passive diffusion of macromolecules smaller than ∼40 kDa (Görlich and Kutay, 1999; Li et al., 2016; Tingey and Yang, 2022) yet requires the assistance of a nuclear transport receptor for the import of macromolecules larger than the ∼40 kDa barrier (Li et al., 2021a; Junod et al., 2023a). Interestingly, regulation such as that observed in the NPC appears to occur in the transition zone of the primary cilium with a selectively permeable barrier of ∼40–70 kDa being reported for the transition zone of the cilium (Kee et al., 2012; Breslow et al., 2013). The presence of a size specific exclusionary barrier is not the only similarity between the NPC and the transition zone, indeed recent studies have identified a wide variety of nucleoporins (Nups), the proteins that together form the super-structure of the NPC and imbue it with function, are also present in the cilium (Table 1). As a result of the presence of Nups in the transition zone of the cilium, it has been proposed that these Nups form the core of a ciliary pore complex (CPC) in a manner similar to that of the NPC (Takao and Verhey, 2016; Endicott and Brueckner, 2018). A proposal that is supported by the observation that deletion of Nup98 increases the ciliary size-exclusion limit by ∼30 kDa (Endicott and Brueckner, 2018; Moran et al., 2024). Nevertheless, it is essential to highlight that, unlike the NPC, the Nups at the base of the cilium are not organized in rings, and the active transport function observed in the NPC is not present in the cilium. This suggests that the barrier function performed by the CPC does not necessitate the organizational structure of an NPC-like pore (Del Viso et al., 2016; Moran et al., 2024).

**TABLE 1:** Nups localized to the cilium.

Nup Name	NPC location	Observed in the cilium basal body	Observed during ciliogenesis	Observed at the ciliary gate	Cell type evaluated	Special observations	References
Nup214	Cytoplasmic filaments	Yes	No	No	hTERT RPE	–	Kee et al., 2012
Nup205	Inner ring	Yes	No	No	hTERT RPE	Deletion disrupted cilia number and length	Braun et al., 2016; Blasius et al., 2019
Nup188	Inner ring	Yes	Yes	No	hTERT RPE	Rare copy number variations	Del Viso et al., 2016; Blasius et al., 2019
Nup160	Outer ring	Yes	No	No	hTERT RPE	–	Kee et al., 2012; Braun et al., 2018
Nup155	Inner ring	Yes	No	No	hTERT RPE	–	Blasius et al., 2019
Nup153	Nuclear basket	Yes	No	No	hTERT RPE	Associated with congenital heart disease	Kee et al., 2012
Nup133	Outer ring	Yes	No	No	hTERT RPE	–	Braun et al., 2018; Blasius et al., 2019
Nup107	Outer ring	Yes	No	No	hTERT RPE	–	Miyake et al., 2015; Braun et al., 2016; Rosti et al., 2017; Blasius et al., 2019
Nup98	Central ring	Yes	Yes	Yes	NIH-3T3	Nup98 regulates cilia length and gating mechanism	Endicott and Brueckner, 2018
Nup93	Linker	Yes	No	No	hTERT RPE	Homozygous missense mutations	Kee et al., 2012; Del Viso et al., 2016; Blasius et al., 2019
Nup88	Linker	Yes	No	No	hTERT RPE	–	Kee et al., 2012
Nup85	Outer ring	Yes	Yes	No	NIH-3T3	Nup85 is required for Nup98 to localize to the ciliary base	Kee et al., 2012; Braun et al., 2018; Endicott and Brueckner, 2018
Nup62	Central ring	Yes	No	Yes	hTERT RPE/ NIH-3T3	–	Kee et al., 2012; Takao et al., 2014; Takao et al., 2017; Blasius et al., 2019
Nup43	Outer ring	Yes	No	No	hTERT RPE	–	Blasius et al., 2019
Nup37	Outer ring	Yes	No	No	hTERT RPE	–	Kee et al., 2012
Nup35	Inner ring	Yes	No	No	hTERT RPE	–	Kee et al., 2012; Blasius et al., 2019
POM121	Luminal ring	Yes	No	No	hTERT RPE	–	Kee et al., 2012

An exhaustive list of nucleoporins (Nup) that have been observed in the cilium. This includes a description of where, or when as appropriate, these Nups have been observed. Further, the cell type in which the Nups were observed and any special observations made in association with the specific Nup.

While the Nups do not organize into an NPC-like pore at the base of the cilium, there certainly appears to be a role for Nups in regulating the transport of macromolecules through the transition zone. Nup62, a phenylalanine-glycine repeat containing Nucleoporin (FG-Nup), critical to forming the selectively permeable barrier in the central channel of the NPC also localizes at the base of the cilium (Kee et al., 2012). To evaluate the impact of this FG-Nup on the import of macromolecules to the cilium, Takao et al. (2014) developed an assay causing forced dimerization of Nup62, the result of which inhibits transport through the NPC (Strambio-De-Castillia et al., 2010), and applied it to the evaluation of ciliary import. Upon applying the Nup62 forced dimerization assay to the primary cilium, attenuation of the entry of cytosolic proteins, but not membrane proteins, was observed (Takao et al., 2014). Suggesting that Nup62 was involved in regulating transport of soluble proteins through the transition zone, but not membrane proteins. Intriguingly, through their bimolecular fluorescence complementation (BiFC) assay, Takao et al. demonstrated that Nup62 has a physical interaction with NPHP4 (Takao et al., 2017), a protein that localizes specifically to the transition zone (Omran, 2010; Shiba and Yokoyama, 2012; Garcia-Gonzalo and Reiter, 2017), as well as the IFT motor kenisin-2 (Takao et al., 2017). To evaluate what other Nups were actively associating with the NPHP complexes in the transition zone Blasius et al. (2019) utilized directed yeast two-hybrid assay to demonstrate that the NPHP complex appears to function as binding partners for several Nups; Nup205, Nup160, Nup85, Nup88, Nup155, Nup133, and Nup43. An observation that is of some interest as it has been observed that loss of NPHP4 weakens and permeabilizes the transition zone barrier resulting in dysregulation of both membrane and soluble protein composition (Jauregui et al., 2008; Williams et al., 2011; Awata et al., 2014; De-Castro et al., 2021).

While the majority of studies have reported a selectively permeable barrier of ∼40–70 kDa, it is notable that one study reports that larger molecules are capable of crossing the barrier. Specifically, Lin et al. (2013) report that they probed the influx of fluorescent proteins via a rapamycin trap assay that permitted the passive diffusion of macromolecules into the cilium but prevented those molecules from diffusing out of the cilium. Using this method, Lin et al. observed the diffusion of particles up to 650 kDa in size diffusing into the interior of the cilium. The results of their study demonstrate that passive diffusion of very large complexes, up to 650 kDa (7.9 nm in diameter), were capable of diffusing into the cilium albeit at a very slow rate over a 60-min assay leading to the proposal that the transition zone permeable barrier functioned as a molecular sieve (Lin et al., 2013). One possible explanation for different studies reporting different barrier cutoff sizes is that the barrier architecture and kinetics differ between discrete ciliary subtypes (Moran et al., 2024). This explanation is supported by the observation that relatively large macromolecules of ∼80 kDa rapidly diffuse across the barrier into the body of the cilium in photoreceptor cells (Najafi et al., 2012), indicating that different cilium types may have different barrier architectures and kinetics.

While the exact nature of the selectively permeable barrier in the transition zone remains a point of experimental interest without a definitive conclusion, it is widely acknowledged that the barrier is regulated in some way. This is supported by the discovery that some ciliary proteins alter localization in response to extracellular clues. For instance, the entry and exit of the proteins Patched, Smoothened, and GPR16 from the cilium are controlled by the hedgehog ligand (Nozawa et al., 2013; Takao and Verhey, 2016; Gigante and Caspary, 2020). This indicates that not only does the barrier discriminate based upon the size of the macromolecule, there also exists a mechanism for selective import and export across the transition zone.

## 3 Cilium localization sequences

The transport and regulation of specific cargoes across the transition zone into the cilium requires a mechanism of targeting toward the cilium. Similar to how proteins destined for the nucleus contain a nuclear localization sequence (NLS) that recruits importin α/β, ciliary localization sequences (CLS), also known as ciliary targeting sequences, have been identified. The first CLS was identified for the kinesin-2 motor KIF17 (Dishinger et al., 2010; Kee et al., 2012; Funabashi et al., 2017), but many more were soon identified. Interestingly, while many CLSs have been reported, they do not share sequence similarity (Long and Huang, 2019). For example, the CLSs for PKD2, Rhodopsin, and CNGB1 proteins contain Vxp motifs (Deretic et al., 1998; Geng et al., 2006; Wang et al., 2012; Long and Huang, 2019); while GPCRs contain (V/I)KARK and Ax(S/A)xQ motifs (Berbari et al., 2008a,b); and INPP5E contains an FDRELYL motif at positions 609–615 (Humbert et al., 2012; Long and Huang, 2019). Each CLS containing macromolecule is recognized by specific membrane protein carriers, which then transport their cargo to the cilia (Long and Huang, 2019). As a result, there is no universal targeting sequence to the cilium, rather a series of specific signals that are recognized by specific carriers. While several cargoes have been identified as containing CLS, the lack of homogeneity makes large scale bioinformatic screening difficult. Therefore, the evaluation of carriers and potential adaptors in ciliary membrane protein trafficking remains a critical area of future research (Long and Huang, 2019). Further, the lack of homogeneity in CLS signals and carriers makes it unlikely that this mechanism is universally responsible for selective import of macromolecules into the cilium.

## 4 Cilium transport routes

Ciliogenesis and homeostatic function of primary cilium require the import of a number of macromolecules significantly larger than the ∼40–70 kDa size limit for passive diffusion. Therefore, a pathway must exist by which proteins are able to bypass this limit via a method reminiscent of facilitated diffusion in the NPC. Indeed the primary cilium makes use of multiple import routes. First, vesicle fusion outside of the primary cilium followed by import into the primary cilium after associating with an IFT train; and second, vesicle fusion at an unknown location inside the primary cilium (Figure 1B). Until recently, the model for all import into the primary cilium involved these two methods, with IFT trains utilizing the pathway peripheral to the axoneme microtubules and vesicle transport being somewhat mysterious, albeit assumed to fuse with the membrane near the periciliary base facilitated membrane diffusion of ciliary transmembrane proteins. Detailed within this section are recent observations that indicate the vesicle facilitated transport pathway is much more complex than the current model depicted in Figure 1B.

### 4.1 Super-resolution methods for studying ciliary transport routes

The structural determination of the cilium has been facilitated using a combination of electron microscopy and super-resolution methods. These methods are exceptional for structural studies as electron microscopy and stochastic optical reconstruction microscopy (STORM) have a spatial resolution of 0.5–4 nm (Lyu et al., 2023) and ∼10 nm (Huang et al., 2008; Xu et al., 2017), respectively. These studies, many of which are highlighted in previous sections, inform as to the shape, form, and structural elements of cilia. While electron microscopy provides unparalleled spatial resolution, it suffers from two key weaknesses. Specifically, sample preparation difficulties and temporal resolution (Turk and Baumeister, 2020; Weissenberger et al., 2021). While many of the sample preparation issues can be ameliorated by optimizing sample preparation, the static nature of the images are inherent to electron microscopy. As a result, this technique is unsuited for deriving dynamic information.

Similar to EM studies, STORM microscopy has been employed to great effect to study the structure of the cilium. STORM, as with all super-resolution methods, relies upon the principle of separation. Specifically, the point spread function (PSF) of stimulated fluorophores must be sufficiently separated, either temporally or spatially, from other emitting PSFs that they may be fit with a gaussian distribution to localize the centroid of the PSF (von Diezmann et al., 2017). The STORM method generates temporal distance between fluorophores through stochastic blinking that is then reconstructed to provide a comprehensive picture of the labeled structures (Rust et al., 2006). A recent study detailing the structure of the transition zone in tetrahymena cilia demonstrated the utility of STORM to great effect (Hazime et al., 2021). Herein, Hazime et al. (2021) demonstrated that transition zone labeled proteins localize to a narrow region of approximately 30 nm, confirming observations made in electron microscopy where the y-links were utilized as markers for the transition zone. Intriguingly, this study found that the IFT train docks approximately 80 nm proximal to the transition zone, near the basal body. Such a docking position indicates that there is a pre-selection process that occurs prior to entry to the transition zone. This data provides valuable insight into the localization and conformation of proteins of interest, however, as with electron microscopy, this technique often requires fixed samples. As a result, dynamic information is the result of inferred averages from aggregations of proteins.

As cilium are intrinsically dynamic structures, evaluating their behavior and structure as a dynamic structure is preferred as it provides a more complete understanding of the processes involved in transition zone transport. To this end, researchers employed stimulated emission depletion (STED) microscopy. This method makes use of a donut shaped laser to limit the excitation area while forcing surrounding fluorophores to the ground state (Leutenegger et al., 2010). By optically engineering the excitation region of the laser, researchers are able to achieve super-resolution localization of ∼50 nm (Hanne et al., 2015). While this resolution is certainly less attractive than that provided by STORM, the temporal resolution of STED as well as the ability to employ this technique in dynamic systems compensates for the issue. Specifically, STED is limited temporally by only two factors; first, the scanning speed and second, the signal to noise ratio. As a result, this technique is capable of providing significant dynamic information about the transport and dynamics within the transition zone. To this end, Lambacher et al. (2016) made use of STED to demonstrate that TMEM-107 is a key protein in Joubert Syndrome in nematodes. Specifically, they found that TMEM-107 recruits the ciliopathy proteins MKS-1, TMEM-231, and TMEM237 where they organize to the membrane periphery and play a key role in diffusion barrier formation. This study provided valuable information regarding the pathology of both Mekler-Gruber and Joubert Syndrome. However, STED is not without limitations in this regard. Specifically, as was demonstrated in previous STORM studies, the width of the transition zone is ∼30 nm (Hazime et al., 2021). Without the addition of stochastically blinking fluorophores, STED is limited to ∼50 nm localization resolution. As a result, the dynamic information presented could be further enhanced.

**FIGURE 2:**
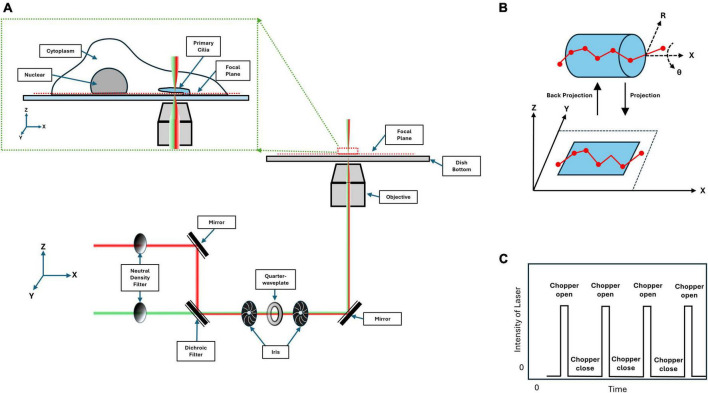
Single-point edge excitation sub-diffraction (SPEED) microscopy light path diagram. **(A)** Depicted here is the light path for SPEED microscopy, showing a 561 nm (red) and 488 nm (green) lasers being focused into a single point at the focal plane. The inset depicts a cartoon of a cilia presenting cell being imaged at the bottom of the cell. **(B)** Shown here is the passage of a fluorescing protein through the rotationally symmetric cylinder of the transition zone. The projection highlights that the 3D information is convolved into 2D when captured by the camera. **(C)** A laser power diagram depicting the addition of an optical chopper with a stimulation ratio of 1:10 on to off to prevent photobleaching.

With this in mind, single-point edge excitation sub-diffraction (SPEED) microscopy was employed. This method achieves PSF separation via spatial distance by controlling the concentration of fluorophores present within the illumination area, thereby achieving single-molecule localization at a specific region of the cilium; namely the transition zone of the cilium (Figure 2A; Li et al., 2021b). This method is capable of achieving localization precision analogous to STORM at ∼10 nm, while being performed in live cells at a temporal resolution of up to 0.4 ms. Unique to this method is a post-imaging 2D to 3D transformation in which the 2D single-molecule localizations captured within a rotationally symmetric structure, such as the primary cilium, are fit to an area matrix and deconvolved to provide a time resolved probability density providing the most probable localization for a given fluorophore within 3D space (Figure 2B; Ruba et al., 2019). This technique, as with any other, has its limitations. Specifically, potential photobleaching of transporting labeled proteins before completing their transport. This can be ameliorated with the addition of an optical chopper to prevent pre-photobleaching (Figure 2C), but researchers should be cognizant of the photostability of fluorophores when designing experiments. Further, the 2D-to-3D transformation is a virtual 3D probabilistic density. Meaning that there is no discrete 3D information for individual points. Further, as this transformation is probability based, it only provides the most dominant 3D routes and may potentially overlook less dominant localizations. By applying this technique to study the transport of macromolecules through the transition zone, a novel transport pathway was discovered that is apparently independent from the intraflagellar transport pathway.

### 4.2 Intraflagellar transport

Intraflagellar transport trains were first termed “rafts” after their initial discovery between the ciliary membrane and microtubules doublets of *C. reinhardtii* cilia by Kozminski et al. (1993) and Webb et al. (2020). After the initial discovery, Kozminski et al. (1995) further interrogated the function of these rafts via correlative light and electron microscopy and found them to be vehicles driving intraflagellar transport rather than inert rafts. These IFT trains are the primary force driving import and export into and out of the cilium. Each train is composed of two large complexes, IFT-A and IFT-B which weigh ∼0.8 and 1 MDa, respectively (Wingfield et al., 2017; Webb et al., 2020). In addition to the megadalton A and B complexes, each train contains the motor proteins required for movement, this includes heterotrimeric kinesin-2 for anterograde transport (in the direction of the ciliary tip) and IFT-dynein for retrograde transport (in the direction of the basal body) (Piperno and Mead, 1997; Cole et al., 1998; Pazour et al., 1999; Lechtreck, 2022). Lastly, trains associate with ciliary proteins that attach to the complex to facilitate import or export (Lechtreck, 2015). It should be noted that the interactions between the IFT train complex members and ciliary protein cargoes appears to be relatively weak, as it has been reported that these complexes readily dissociate when isolated from the cilium (Piperno and Mead, 1997; Rompolas et al., 2007; Craige et al., 2010; Mencarelli et al., 2013; Wingfield et al., 2017).

Intraflagellar transport train transport can be most simply broken into five discrete stages. In the first stage, IFT trains assemble at the base of the cilium near the transition fibers where IFT-A/B and their motor proteins associate. Cryo-electron tomography indicates that IFT-B functions as the backbone for the complex (Jordan et al., 2018), an observation that has been supported by experimental evidence that IFT trains cease to function when IFT-B is knocked out (Perkins et al., 1986; Cole et al., 1998; Pazour et al., 2000). In stage two the now assembled IFT train crosses the diffusion barrier at the transition zone. It remains unknown exactly how the IFT train overcomes this barrier. Indeed, cryo-electron tomography of *Chlamydomonas* cilium indicates that the typical IFT train is ∼50 nm in diameter (Jordan et al., 2018), and is therefore similar in width to the microtubule doublet. It is unknown whether the IFT train undergoes conformational changes to pass through the transition zone, but the unique shape of Y-links have been implicated as potentially being involved due to their positioning at a point that would normally interrupt anterograde transport (Garcia-Gonzalo and Reiter, 2017; Webb et al., 2020). Despite the lack of granular detail on the mechanism of transport through the transition zone, it is clear that IFT train transport through the transition zone is coupled with transition zone integrity as mutations in IFT-A and dynein-2 subunits perturb the localization of transition zone proteins (Jensen et al., 2018; Scheidel and Blacque, 2018; Webb et al., 2020).

Stage three is anterograde transport of the IFT train to the ciliary tip where the IFT trains move along axonemal microtubules to deliver tubulin (Hao et al., 2011), axonemal precursors (Qin et al., 2004), receptor ion channels (Qin et al., 2005) and other cargoes to the distal tip of the cilium. Anterograde transport of the IFT train is powered by a heterotrimeric kinesin-II (also called Kif3) (Engelke et al., 2019). The heterotrimer is composed of Kif3A, Kif3B, and Kap3. Both Kif3A and Kif3B contain motor domains on the N-terminus as well as coiled-coil segments that mediate heterodimerization. Kap3 differs significantly from Kif3A/B as it contains a series of hydrophobic Armadillo repeats, a region that recognizes charged or hydrophobic residues. This is of some interest as Armadillo repeats are a feature of nuclear importin-α, which is formed of consecutive armadillo repeats that interact with FG-Nups, Such as Nup62, within the NPC to facilitate the transport of cargoes (Görlich et al., 1994; Aramburu and Lemke, 2017). The presence of the Armadillo repeats present in kinesin-II may explain the observation by Takeo et al. that Nup62 interacts directly with kinesin-II (Takao et al., 2017). Further, Kap3 was found to be required for *in vivo* transport, but not *in vitro*. When combined with the observed interaction with Nup62, it is possible that Kap3 helps to facilitate regulation of transport through the barrier (Mueller et al., 2005). It is particularly interesting how the IFT train avoids having a tug-of-war between the kinesin motor and dynein motor during transport. Recent cryo-EM imaging found that during anterograde transport the dynein motor is inactivated via positioning. Specifically, the dynein motor is also held away from the tubulin track, thereby inhibiting any errant binding and ensuring uni-directional transport during anterograde transport (Jordan et al., 2018).

Stage four occurs when the IFT train arrives at the distal tip of the cilium. Upon arrival at the distal tip, IFT trains release their cargo and undergo major remodeling to pick up new cargo and facilitate retrograde transport (Chien et al., 2017). Interestingly, it was observed using single-molecule microscopy that IFT trains pause for only a few seconds upon arrival at the ciliary tip (Mijalkovic et al., 2018), indicating that the process of remodeling occurs rapidly. The primary change at this stage, beyond the change in cargoes, is that the kinesin motor that drives anterograde transport is inactivated and the dynein motor facilitating retrograde transport is activated (Chien et al., 2017; Webb et al., 2020). As was discussed previously, the dynein motor is positioned away from the microtubule track during anterograde movement, therefore significant remodeling is required to move the dynein motor into a functional position. Due to the speed and transient nature of this very quick process, relatively little is known about the specific granular interactions taking place at the ciliary tip (Mijalkovic et al., 2018). The fifth and final stage is the retrograde transport of the IFT train back toward the basal body and through the transition zone. During retrograde transport, kinesin is inactivated and the IFT-dynein motor, Dynein-2, is activated. With Dynein-2 active, the complex then returns to the ciliary base by moving along the A-tubule (Stepanek and Pigino, 2016; Jordan et al., 2018; Webb et al., 2020).

Intraflagellar transport signaling makes use of an adaptor complex to facilitate membrane protein trafficking, the BBSome. This complex was discovered in 2007 (Nachury et al., 2007; Loktev et al., 2008) by researchers interrogating the molecular basis of the genetically heterogenous recessive Mendelian disorder Bardet-Biedl syndrome (BBS), a syndrome characterized by polydactyly, genital malformation, retinal degeneration, morbid obesity, and kidney anomalies (Wingfield et al., 2018). The results of this study was the identification of an octameric complex consisting of BBS1, BBS2, BBS4, BBS5, BBS8, BBS9, BBS18/BBIP10 and the small GTPase BBS3/Arl6 (Nachury et al., 2007; Loktev et al., 2008; Jin et al., 2010; Scheidecker et al., 2014). This complex functions as a cargo adaptor for IFT and can therefore be considered an expansion upon the canonical IFT train.

The BBSome complex has been proposed to assemble in a stepwise fashion at pericentriolar satellites, nucleating around BBS4. The final component added, BBS1, then mediates translocation to the ciliary base (Prasai et al., 2020). The fully assembled BBSome then associates with the IFT train and proceeds through anterograde transport until arriving at the ciliary tip. During remodeling for retrograde transport from the ciliary tip, BBSome complexes recognize activated GPCRs and form a BBSome-coat. BBS1 has been implicated as the critical component for cargo recognition and is likely the region associating with active GPCRs (Seo et al., 2009, 2011; Jin et al., 2010; Ruat et al., 2012; Zhang et al., 2012; Bhogaraju et al., 2013; Su et al., 2014; Klink et al., 2020). The BBsome-coated active GPCRs are then then picked up by IFT trains for retrograde transport (Wingfield et al., 2018). The current specific mechanism for association between the BBSome and IFT trains remains somewhat nebulous, however, it has been reported that both IFT25 and IFT27 knockout mutants result in accumulation of BBSome and BBSome cargoes at the ciliary tip (Keady et al., 2012; Eguether et al., 2014), suggesting that these IFT proteins may mediate BBSome binding for retrograde transport in some way.

As with the other components of IFT, the specific mechanisms of cargo selectivity for the BBSome complex remain unclear. Interestingly, many BBSome subunits have been reported to share structural elements of the COPI, COPII, and clathrin-adaptor complexes (Nakayama and Katoh, 2017). BBS4 and BBS8 specifically contain sequence similarity to the COP-ε subunits of vesicle-coating complexes (Jin et al., 2010). This role of BBsome subunits in vesicle coating has been suggested to contribute to the lateral transport of ciliary membrane proteins rather than any involvement in vesicle trafficking into the cilium (Wingfield et al., 2018). However, it has been reported that in *T. brucei* to localize to the flagellar pocket and to adjacent cytoplasmic vesicles (Langousis et al., 2016). Suggesting that the BBsome may have an expanded role in vesicle trafficking.

In the majority of cells, IFT trains run continuously, in addition to their role in signaling, it has been proposed that cilia and their axonemes are inherently unstable and therefore require constant transport of building materials in order to maintain their appropriate length (Marshall and Rosenbaum, 2001; Lechtreck et al., 2017). It is therefore interesting to note that the IFT cycle in *C. reinhardtii* and *T. thermophila* both exhibit a “semi-open” system, where IFT-B proteins from retrograde trains immediately queue for release back into the cilium in different stages of assembly (Wingfield et al., 2017). If this system is shown to be present in mammalian cells as well, it could indicate that transport into the cilium via IFT trains and their co-factors could be more complex than the current model indicates.

### 4.3 Vesicle-related transport

The vesicle mediated transport pathway first arose due to the occasional appearance of vesicle-like structures inside primary cilia and photoreceptors (Reese, 1965; Wakefield and Waite, 1980; Poole et al., 1985; Jensen et al., 2004; Shah et al., 2008; Arellano et al., 2012; Chuang et al., 2015; Banks et al., 2018; Jana et al., 2018). This model was first proposed largely as a derivation of a study on frog photoreceptors that demonstrated that rhodopsin localizes to vesicles that appeared to be fusing at the base of the photoreceptor’s connecting cilia (Papermaster et al., 1985). However, the observation of vesicles within the primary cilium (Reese, 1965; Wakefield and Waite, 1980; Poole et al., 1985; Jensen et al., 2004; Shah et al., 2008; Arellano et al., 2012; Chuang et al., 2015; Banks et al., 2018; Jana et al., 2018) resulted in a model that accomplishes transport and enrichment of ciliary proteins in a single step. In brief, this model proposes that cargo bearing vesicles fuse at an unknown location within the perimeter of the membrane diffusion barrier of primary cilia, thereby delivering cargo and enriching the primary cilium with ciliary proteins (Figure 1B; Hunnicutt et al., 1990; Vieira et al., 2006; Pazour and Bloodgood, 2008; Nachury et al., 2010; Chuang et al., 2015; Jensen and Leroux, 2017).

This model requires the cooperation of several vesicle associated proteins and has differing degrees of activity with vesicle mediated transport being very active during ciliogenesis and less active during the stable state of the cilium (Westlake et al., 2011; Zhao et al., 2023). Vesicle mediated transport to the cilium is well documented during ciliogenesis, as far back as 1962 vesicle like structures were detected in the early stages of ciliogenesis associating with the mother centriole (Sorokin, 1962). Subsequent studies have expanded upon this role for vesicular transport and elucidated a signaling pathway where Rab11 traffics Rabin8 on a periciliary vesicle where it activates Rab8 and subsequently mediates vesicle transport either to the distal appendage or to the subdistal appendage. This signaling cascade and direct transport of cargo via vesicles during ciliogenesis has been reviewed in great detail in a well-written, recently published review (Zhao et al., 2023).

**FIGURE 3:**
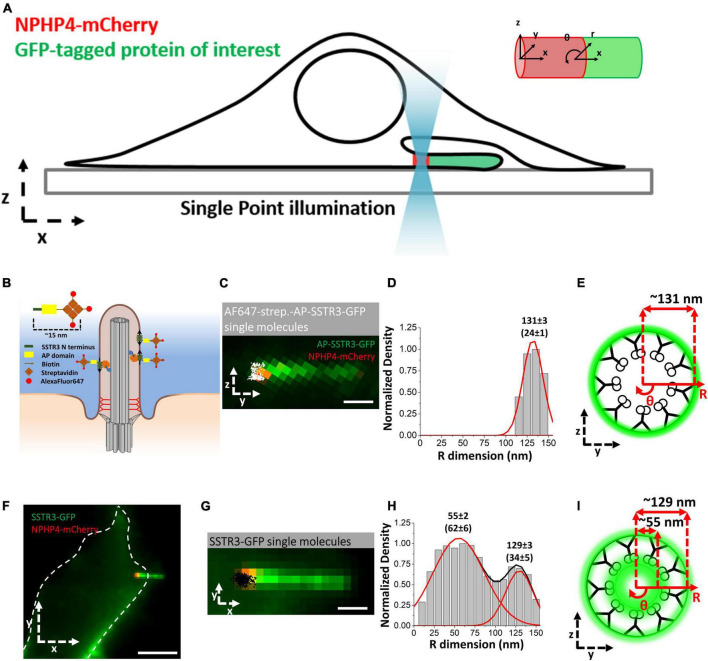
Single-Molecule Super-Resolution Imaging of labeled SSTR3 in primary cilia. **(A)** Schematic of SPEED microscopy–based imaging of labeled proteins moving through the TZ labeled with NPHP4-mCherry. The spatial distributions of transiting molecules are described by both Cartesian (x, y, z) and cylindrical (x, θ, r) coordinate systems. **(B)** Schematic of the external labeling procedure. The extracellular SSTR3 N-terminus (green) is tagged with the AP (yellow) and biotinylated (arrow) for binding to AlexaFluor647 (red)-labeled streptavidin (orange). **(C)** Image of primary cilium in live cells co-expressing AP-SSTR3-GFP (green) and NPHP4-mCherry (red) overlaid with two-dimensional single-molecule Alexa-Fluor-647 externally labeled SSTR3 locations (white). Scale bar: 1 μm. **(D)** Single-molecule AP-SSTR3-GFP locations in the TZ plotted along the R dimension in the cylindrical system. **(E)** Spatial representation of the histogram in **(D)**. **(F–I)** Imaging of SSTR3-GFP. Data reprinted with permission from the American Society for Cell Biology (Ruba et al., 2023).

A recent study has found that the vesicle mediated transport route is not limited in scope but continues throughout the life cycle of the primary cilium and contributes to the import of transmembrane G protein-coupled receptors SSTR3 and HTR6. Using the high-speed super-resolution microscopy method Single-Point Edge-Excitation sub-Diffraction (SPEED) microscopy, Ruba et al. (2023) collected single-molecule trajectories of labeled proteins at 10–20 nm localization precision at a capture rate of 2 ms. This technique is unique as with a sufficiently high temporal and spatial resolution, single-molecule data collected within a rotationally symmetrical structure such as the NPC or primary cilium are able to be reconstructed in three-dimensions (Ruba et al., 2018, 2019; Li et al., 2021b; Junod et al., 2023b; Tingey et al., 2023), providing researchers with the relative 3D probability density of the proteins of interest. To this end, an NIH-3T3 cell line stably expressing NPHP4-mCherry was imaged to localize the transition zone and evaluate the 3D transport routes of cargoes into and out of the primary cilium (Figure 3A).

In this study, Ruba et al. (2023) tracked the single molecule dynamics of SSTR3 in NIH3T3 cells. To this end, a novel labeling strategy was developed permitting researchers to differentiate between membrane bound SSTR3 and unbound SSTR3 within primary cilium at different time points (Figure 3B). This was accomplished through the transfection of an SSTR3 cassette containing an N-terminal AP domain and a C-terminal GFP. The AP domain is an acceptor peptide that, when expressed with the biotin ligase BirA, becomes biotinylated following translation. The biotinylated AP-SSTR3-GFP is then transported to the cilium where it reaches its final location in the ciliary membrane. When streptavidin labeled with Alexa Fluor 647 (AF647) is introduced, the extracellular AP domain binds to the streptavidin, thereby labeling the membrane bound population of SSTR3 in a highly specific manner (Figure 3C; Ruba et al., 2023). The results of this experiment yielded anticipated results, where SSTR3 was localized only to the ciliary membrane at the transition zone (Figures 3D, E). adjacent to the measured localizations of IFT components IFT20 and IFT43 (Ruba et al., 2023).

When this same experiment was performed transfecting with an SSTR3 cassette containing GFP on the C-terminus, a completely unanticipated result was discovered (Figures 3F, G). Specifically, that there were two discrete populations of SSTR3 within the transition zone of the cilium. This population consisted of membrane bound SSTR3, as was identified in the earlier experiment, and a population of SSTR3 with a density peak at 55 ± 2 nm on the R-dimension (Figures 3H, I). This peak corresponds to the ciliary lumen along the interior wall of the microtubule doublets. The presence of SSTR3 within the lumen of the cilium was both unanticipated and significant, as this indicated the presence of a previously uncharacterized transport pathway.

**FIGURE 4:**
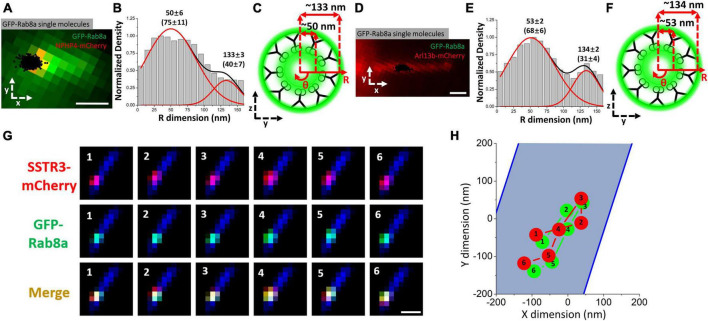
The vesicle associated protein Rab8a transports in close association with SSTR3. **(A–C)** Imaging of GFP-RAB8A in the TZ. **(A)** Epifluorescence microscopy image of GFP-RAB8A (green) and NPHP4-mCherry (red) overlaid with single-molecule GFP-RAB8A locations (black). Scale bar: 1 μm. **(B)** Single-molecule GFP-RAB8A locations plotted along the R dimension. **(C)** Spatial representation of B. **(D–F)** Imaging of GFP-RAB8A in the cilium shaft. **(D)** Epifluorescence microscopy image of GFP-RAB8A (green) and Arl13b-mCherry (red) overlaid with single-molecule GFP-RAB8A locations (black). **(E)** Single-molecule GFP-RAB8A locations plotted along the R dimension. **(F)** Spatial representation of **(E)**. **(G)** Frame by frame images of single molecule images of SSTR3-mCherry and GFP-Rab8a within the transition zone. **(H)** A plot of the single frame images depicted in **(G)**. The blue area represents the body of the cilium, the red indicates SSTR3, and the green indicates Rab8a. Scale bar: 1 μm. Data reprinted with permission from the American Society for Cell Biology (Ruba et al., 2023).

Recognizing that Rab8a promotes docking of vesicles at the basal body of the cilium (Yoshimura et al., 2007; Nachury et al., 2010; Zhao et al., 2023). Ruba et al. (2023) hypothesized that a possible explanation for the novel transport pathway observed for SSTR3 is that Rab8a was facilitating the transport of SSTR3. This was subsequently supported by imaging GFP-Rab8a at both the transition zone (Figures 4A–C) and the axoneme of the primary cilium concomitantly with SSTR3 (Figures 4D–F). The results of this imaging depicted a similar transport route to that observed in single-molecule SSTR3 tracking as well as clear co-movement between the Rab8a and SSTR3 (Figures 4G, H). The close association between the two suggests an association between SSTR3 and Rab8a in transport, further the presence of the pathways indicated the possible transport of vesicles through the lumen of the primary cilium, thereby facilitating the transport of G protein-coupled receptors such as SSTR3 into the primary cilium. While the presence of Rab8a at the membrane was anticipated as Rab8a has been reported to interact with the membrane due to its COOH-terminal (Huber et al., 1993; Grigoriev et al., 2011; Westlake et al., 2011), close co-tracking between SSTR3 and Rab8a in both the ciliary lumen and membrane was not.

**FIGURE 5:**
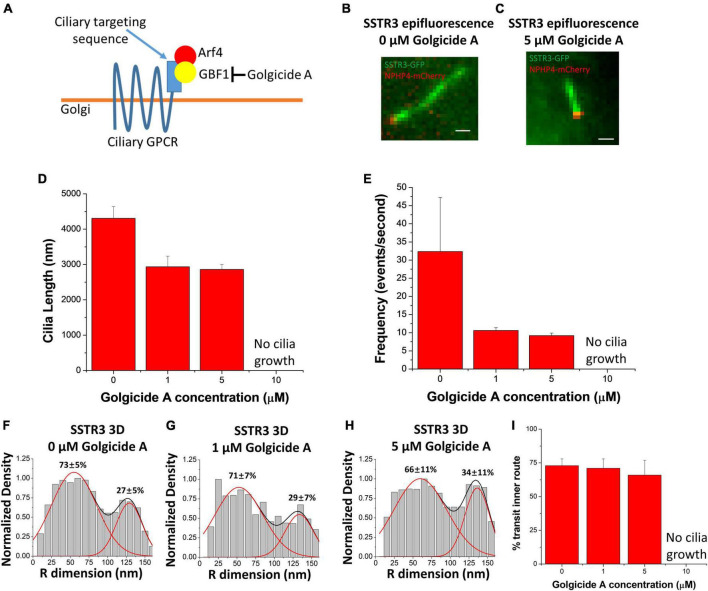
Golgicide A reduces SSTR3 frequency but does not alter transport routes. **(A)** Schematic outlining Golgicide A’s mechanism of action. **(B,C)** Epifluorescence images of SSTR3-GFP in NIH-3T3 cells treated with **(B)**, no Golgicide (**A** or **C**), 5 μM Golgicide A for 24 h. Scale bars: 1 μm. **(D)** Bar graph showing cilia length vs. Golgicide A concentration (0 μM *n* = 29, 1 μM *n* = 8, 5 μM *n* = 12, bar graph represents mean ± SE). **(E)** Bar graph showing SSTR3 single-molecule frequency vs. Golgicide A concentration (0 μM *n* = 5, 1 μM *n* = 3, 5 μM *n* = 4, bar graph represents mean ± SE). **(F–H)** Three-dimensional transport routes for SSTR3 in 0, 1, and 5 μM Golgicide A, respectively, plotted along the R dimension. **(I)** Summary of percentages of SSTR3 transport in the inner transport route for 0, 1, 5, and 10 μM Golgicide A. Data reprinted with permission from the American Society for Cell Biology (Ruba et al., 2023).

Having observed the common transport pathway being utilized by both Rab8a and SSTR3, Ruba and colleagues treated cells with Golgicide A (GCA), an inhibitor of Golgi vesicular transmembrane protein transport (Figure 5A). Similar to previous experiments, NIH3T3 cells underwent serum starvation and were supplemented with different concentrations (0, 1, 5, and 10 μM) of GCA to evaluate if the SSTR3 observed within the lumen of the primary cilium was associated with vesicles or engaged in another, as of yet, uncharacterized behavior. If the SSTR3 within the lumen of the primary cilium was engaging in some way with vesicles, as the transport commonality with Rab8a indicated, then the transport pathway of the SSTR3 within the primary cilium would likely change in response. Intriguingly, treatment with GCA resulted in significantly shorter cilium length in the 1 and 5 μM conditions and no cilium growth in the 10 μM condition (Figures 5B–D). The 1 and 5 μM conditions both demonstrated a change in the transport route with a decreased concentration within the lumen but not total removal (Figures 5E–I). Unexpectedly, the treatment of GCA impacted the total frequency of events for both membrane and lumen transport routes, suggesting that a similar path of entry into the cilium is employed by both transport pathways. This is interesting as it has previously been proposed that vesicles bearing membrane bound ciliary proteins fuse at or near the base of the pericilium permitting the diffusion of ciliary transmembrane proteins along the ciliary membrane. This observation suggests that both the lumen and membrane transport routes contain a vesicular transport component.

**FIGURE 6:**
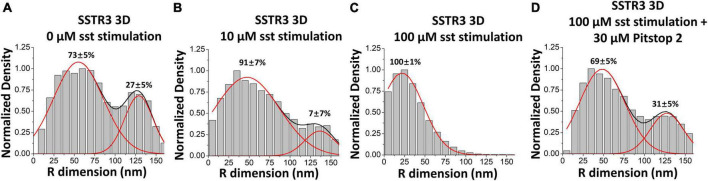
Somatostatin stimulation causes an increase in the luminal transport route of SSTR3. **(A–D)** Three-dimensional transport routes in the TZ for SSTR3 in 0, 10, and 100 μM somatostatin and 100 μM somatostatin with 30 μM Pitstop 2. Data reprinted with permission from the American Society for Cell Biology (Ruba et al., 2023).

After observing the transport pathways of SSTR3 without an imposed directionality on the molecule, Ruba et al. next evaluated the transport pathway of SSTR3 under a directional influence. Previous studies demonstrated convincingly that SSTR3 is actively removed from primary cilia following binding with somatostatin (Green et al., 2016; Ye et al., 2018). The proposed mechanism for SSTR3 removal from the primary cilium involves the recruitment of β-arrestin, which then binds to SSTR3 and promotes clathrin-coated endocytosis (Oakley et al., 1999; Green et al., 2016). Therefore, an experiment was devised where the single-molecule trajectories of SSTR3 at the transition zone would be evaluated under different concentrations of somatostatin (0, 10, and 100 μM) to interrogate the role of clathrin-coated endocytosis in the removal of SSTR3 from the cilium. Interestingly, the transport pathway of SSTR3 demonstrated a marked shift toward the central lumen pathway in the presence of somatostatin (Figures 6A, B). At the highest concentration tested (100 μM) 100% of all observed trajectories were observed to be located to the central luminal pathway (Figure 6C). Next, 30 μM Pitstop 2, a potent inhibitor of clathrin-coated endocytosis (von Kleist et al., 2011), was added to the 100 μM condition (Figure 6D). The addition of Pitstop 2 prevented the preferential shift toward the luminal pathway and returned the pathway composition back to the proportions observed in the 0 μM condition. Together, this strongly suggests that clathrin-coated endocytosis plays a role in facilitating movement through the central luminal route.

Lastly, as has been briefly mentioned above, the vesicular transport model is well documented during ciliogenesis and has been noted as being very active during ciliogenesis and less so during the stable state of the primary cilium (Westlake et al., 2011; Zhao et al., 2023). In agreement with the previous findings, Ruba et al. demonstrated that both SSTR3 and HTR6 preferentially utilize the central luminal pathway during early ciliogenesis and then normalize to the observed proportionality in the stable state, approximately 24 h following the start of ciliogenesis; a timeframe that concurs with previous studies (Westlake et al., 2011; Zhao et al., 2023). Where this study differs is the recognition that following the establishment of the primary cilium (∼24 h) the vesicle pathway does not cease, as has been suggested. Taken together, this data presents further evidence for the vesicle mediated transport of cargoes into the primary cilium and establishes the vesicle mediated transport as a significant transport route for import and export of ciliary associated proteins.

## 5 Perspective

**FIGURE 7:**
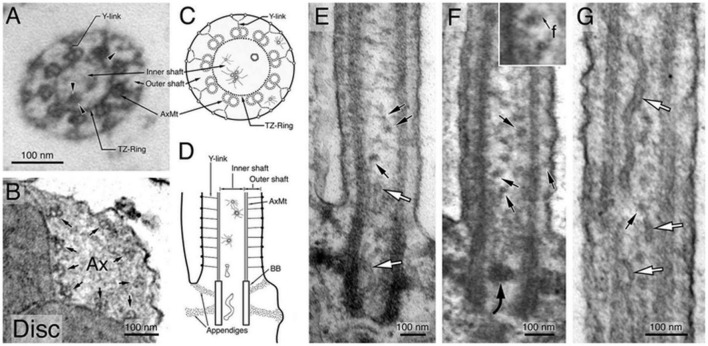
Ultrastructural characterization of membrane structures within the Connecting Cilium (CC) and basal Outer Segment (OS) axoneme. Morphological characterization of the and OS axonemes of untransfected, unlabeled Sprague–Dawley rat rods. Electron micrographs of the representative cross-sectional view of the CC **(A)** and the basal OS axoneme **(B)**. Both Y-link and TZ-ring are prominent in the CC **(A)** but not in the OS axoneme **(B)**. Arrowheads in **(A)** point to small vesicles, present in both inner and outer shafts. Arrows in **(B)** point to the nine AxMt doublets. **(C,D)** are schematic diagrams depicting simplified versions of the electron micrographs of **(A,E)**, respectively. **(E–G)** Representative longitudinal sectional views of the CC. White arrows point to smooth membrane tubules; black arrows point to some of the representative coated small vesicles that often had filament (f) attached [an enlarged view is shown in the inset of **(F)**]. A curved arrow points to a vesicle cluster that happens to be situated in the middle of a basal body. Figure reprinted with permission from Springer Nature (Chuang et al., 2015).

The findings presented by Ruba et al. propose the existence of a heretofore uncharacterized transport pathway involving vesicle-mediated transport through both the lumen and membrane region of the cilium. Although the evidence presented in their study is compelling (Poole et al., 1985), it is not without its challenges, as is common in any study challenging established paradigms. One notable challenge is the question concerning electron microscopy (EM) imaging of vesicles within the primary cilium. There appears to be a discrepancy between the demonstrated presence of Rab8a within the ciliary lumen and the seemingly sparse representation of vesicles in EM images of the primary cilium’s lumen. As was noted earlier, the presence of vesicles within cilia is not a new or novel observation (Reese, 1965; Wakefield and Waite, 1980; Poole et al., 1985; Jensen et al., 2004; Shah et al., 2008; Arellano et al., 2012; Chuang et al., 2015; Banks et al., 2018; Jana et al., 2018). However, their relative observed sparsity may be the result of technology associated issues. For example, it is possible that sample preparation for EM imaging resulted in disruption of unbound vesicles, causing them to wash out of the cilium. Also possible is that the vesicles being imaged are relatively small, with a low electron density. Such a makeup would render them more difficult to image via traditional EM methods. Indeed, small vesicles of a size of <20 nm in size have been reported within the lumen, directly distal to the transition zone, in the outer segment of mammalian rods, a modified cilium (Figures 7A–G; Chuang et al., 2015). This observation led Chuang et al. (2015) to propose the existence of multiple ciliary gate entry pathways in mammalian rod photoreceptors, a concept that has received support from subsequent studies.

Another possible explanation as to the apparent sparsity of vesicles within cilium is the possibility of different cilium having differential quantities of vesicles present in the cilium. Interestingly, the preprint (at the time of writing) of a recent study describing an in-depth cryo-EM analysis of primary cilium on excitatory neurons, inhibitory neurons, astrocytes, and oligodendrocyte precursor cells demonstrated the differential vesicle concentrations in primary cilia of differing cell types (Ott et al., 2023). This study reported that while vesicle presence in astrocyte and neuron cilia was relatively rare, there was a significant portion of the population containing at least a single vesicle. Intriguingly, the OPC cilia that were observed contained a plentiful and diverse population of vesicles with nearly 100% of the *P* > 270 OPC dataset contained vesicles. In contrast to the relatively small (<20 nm) vesicles observed in the interior of the axoneme (Chuang et al., 2015), the observed vesicles in the OPC cilia were of a relative larger size (∼100 nm). Further, Ott et al. (2023) indicated that vesicles were only observed adjacent to the axoneme microtubules on the exterior of the axoneme and are unable to comment on the origin of the vesicles. Specifically, static imaging approaches, such as cryo-EM are limited to a single timepoint and are unable to determine whether the vesicle originated in the cell body and was trafficked into the cilium or if they are the product of endocytosis. Therefore, further analysis using an *in vivo* high-speed super-resolution imaging technique will likely need to be employed to further characterize the nature of these vesicles.

The differential presence of vesicles within different populations of cilia is not overly surprising, as different cilia engage in a variety of specialized functions. It is therefore logical that their discreet composition would be somewhat different. Furthermore, the differential presence of vesicles in varying cilia bearing cells is unsurprising. As was commented on earlier, the makeup and architecture of the transitional zone barrier may differ significantly between cilia bearing cells resulting in a significant variation in size exclusion barrier, cargo import rates, and potentially quantity and location of vesicles within primary cilia. Such an observation may also help to explain the relative scarcity in observed cilium via EM imaging. Perhaps the populations that have previously been imaged contain relatively few or only small vesicles that are not electron dense, and therefore less likely to be imaged. Regardless of the cause, the presence of vesicles within the cilium has been confirmed via EM, Cryo-EM, and super-resolution imaging; both in the lumen and adjacent to the microtubules on the exterior of the axoneme.

**FIGURE 8:**
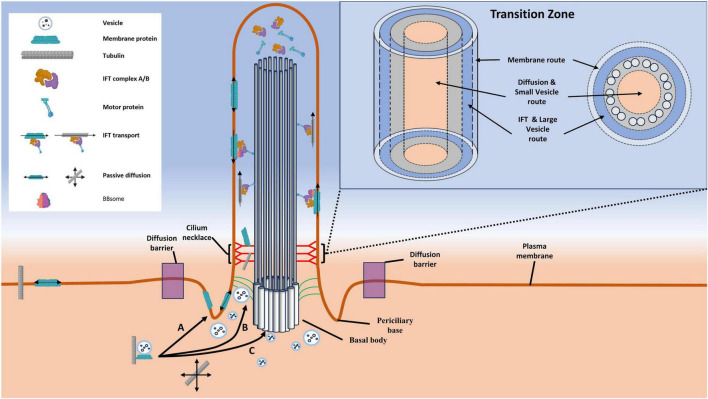
Diagram illustrating transport routes through the transition zone, with a focus on highlighting the Vesicle-Assisted Transport (VAT) model. Shown here are the three discrete transport routes that have been characterized to date. **(A)** The membrane route is utilized by macromolecule bearing vesicles that fuse at a point between the diffusion barrier and the transition zone, permitting their membrane-bound cargoes to diffuse along the ciliary membrane. **(B)** This pathway is utilized by both IFT trains and large vesicles. **(C)** The luminal transport route is preferentially utilized by small vesicles and passively diffusing molecules, although it is likely that both of these may be present in the IFT pathway.

The presence of vesicles near the membrane peripheral to the axoneme is expected, given the findings of Ruba et al., who reported the localization of the vesicle-associated protein Rab8a in both the lumen of the axoneme and the exterior route near the periphery of the axoneme. Distinct vesicle localizations within the primary cilium may suggest the existence of size-specific selectivity in transport routes through the cilium. Smaller cargoes may preferentially use the lumen of the axoneme, while larger vesicles may rely on the exterior pathway traditionally utilized by the megadalton-sized IFT trains. In light of these groundbreaking observations, we propose a novel model to elucidate the transport routes of cargoes through the transition zone of cilia, involving vesicles, referred to as the Vesicle-Assisted Transport (VAT) model. This model, in conjunction with the established IFT pathway, outlines three distinct entryways (and exits as appropriate) for ciliary-bound cargoes (Figure 8): (1) The membrane-bound pathway: As previously detailed, vesicles bearing transmembrane ciliary proteins fuse at or near the periciliary base, allowing the passive diffusion of membrane-bound proteins along the ciliary membrane. (2) The IFT pathway and large vesicle transport pathway occur concomitantly within the same route. This pathway is utilized by large vesicles unable to pass through the size-limited barrier in the lumen of the cilium and may leverage IFT machinery to traverse the transition zone. While EM data indicates a preference for large vesicles in this pathway, the presence of small vesicles cannot be ruled out. (3) The luminal vesicle transport route is utilized by small vesicles and is localized along the interior of the axoneme microtubules. This pathway is also the preferred entrance for small, passively diffusing molecules that traverse the most central region of the lumen. Collectively, the IFT-VAT model of transport pathways describes segregated routes based on transport mechanisms and cargo sizes.

The VAT model of transport pathways holds broad implications for a multitude of biological processes, not only for membrane-bound G protein-coupled receptors (GPCRs) such as SSTR3 and HTR6 but also for other imported macromolecules like the BBSome complex or the contents of ciliary extracellular vesicles. As detailed earlier in this text, BBSome components exhibit structural elements akin to vesicle coat proteins COPI and COPII. Additionally, in T. brucei, the BBSome has been observed to localize to the base of the cilium in a region associated with vesicle-facilitated import. Considering these factors, it is plausible that the anterograde IFT train may not be the sole mechanism of import for BBSomes; rather, a portion might be imported via the lumen of the cilium through a vesicle-mediated pathway. While this remains a captivating possibility, rigorous experimentation is required for validation.

Another intriguing potential aspect of this is the composition and secretion of extracellular vesicles from cilia. In their 2021 publication, Mohieldin et al. (2021b) demonstrated that the protein composition of vesicles secreted from cilium are distinct from those originating in the cytoplasm. Upon further investigation, Mohieldin et al. (2021a) demonstrated that the contents of the ciliary derived extracellular vesicles contain unique biomarkers that have direct protein-protein interactions with proteins associated with known ciliopathies and Alzheimer’s Disease, an observation that is supported by an observed correlation between extracellular vesicle associated proteins, cilia, and neurodegenerative disorders (D’Anca et al., 2019; Alhassen et al., 2021). While a more comprehensive study to determine the specific role of these ciliary derived extracellular vesicles is needed, the implications are intriguing. The current model suggests that vesicles excreted from cilia bud from the tip of the cilium to facilitate waste removal and/or cell-cell communication (Moran et al., 2024). As it is proposed they bud from the tip of the cilium, the composition must therefore be comprised of cargo that has successfully passed through the ciliary selectively permeable barrier. As a result, the potential for a secondary pathway for these proteins to import into the cilium, apart from the IFT pathway, is both intriguing and physiologically relevant as it may present a future target for therapeutic intervention.

## Author contributions

WY: Conceptualization, Data curation, Formal analysis, Funding acquisition, Investigation, Methodology, Project administration, Resources, Software, Supervision, Validation, Visualization, Writing – original draft, Writing – review and editing. MT: Conceptualization, Data curation, Formal analysis, Investigation, Methodology, Project administration, Resources, Software, Supervision, Validation, Visualization, Writing – original draft, Writing – review and editing. AR: Writing – original draft, Writing – review and editing. ZJ: Writing – original draft, Writing – review and editing.
